# Supplementary material for: A Mutation in Mouse MT-ATP6 Gene Induces Respiration Defects and Opposed Effects on the Cell Tumorigenic Phenotype
Source: Int J Mol Sci. 2023 Jan 9;24(2):1300. doi: 10.3390/ijms24021300 (PMC9865613; doi:10.3390/ijms24021300)
Supplement: Supplementary file 1 [file ijms-24-01300-s001.zip › ijms-2108138-supplementary.pdf]

Supplementary materials for article

A Mutation in Mouse MT-ATP6 Gene Induces Respiration Defects and Opposed Effects on the Cell Tumorigenic Phenotype

Raquel Moreno-Loshuertos [1,2,†], Nieves Movilla [3,†], Joaquín Marco-Brualla [1], Ruth Soler-Agesta [1], Patricia Ferreira [1,2], José Antonio Enríquez [4] and Patricio Fernández-Silva [1,2,*]

1 Department of Biochemistry and Molecular and Cell Biology, University of Zaragoza, 50009 Zaragoza, Spain
2 Institute for Biocomputation and Physics of Complex Systems, University of Zaragoza, 50018 Zaragoza, Spain
3 Department of Mechanical Engineering, Multiscale in Mechanical and Biological Engineering (M2BE), Aragon Institute of Engineering Research (I3A), University of Zaragoza, 50018 Zaragoza, Spain
4 Centro Nacional de Investigaciones Cardiovasculares (CNIC), 28029 Madrid, Spain
* Correspondence: pfsilva@unizar.es (P.F-S.); Tel.: +34-976761285
† These authors contributed equally to this work.

Table S1: Identity analysis of *mt-Atp6* DNA and protein sequences between *Mus musculus* and different animal species

Species	Identity (%)	
	Protein	DNA
Mus musculus		
vs Homo sapiens	74.8	71.2
vs Pan troglodytes	75.2	71.5
vs Macaca mulatta	63.3	65.6
vs Canis lupus familiaris	82.7	72.9
vs Bos taurus	78.8	77.1
vs Rattus norvegicus	94.2	85.4
vs Gallus gallus	54.7	62.1
vs Danio rerio	54.2	60.4
vs Drosophila melanogaster	40.2	53.4
vs Anopheles gambiae	39.7	51.9

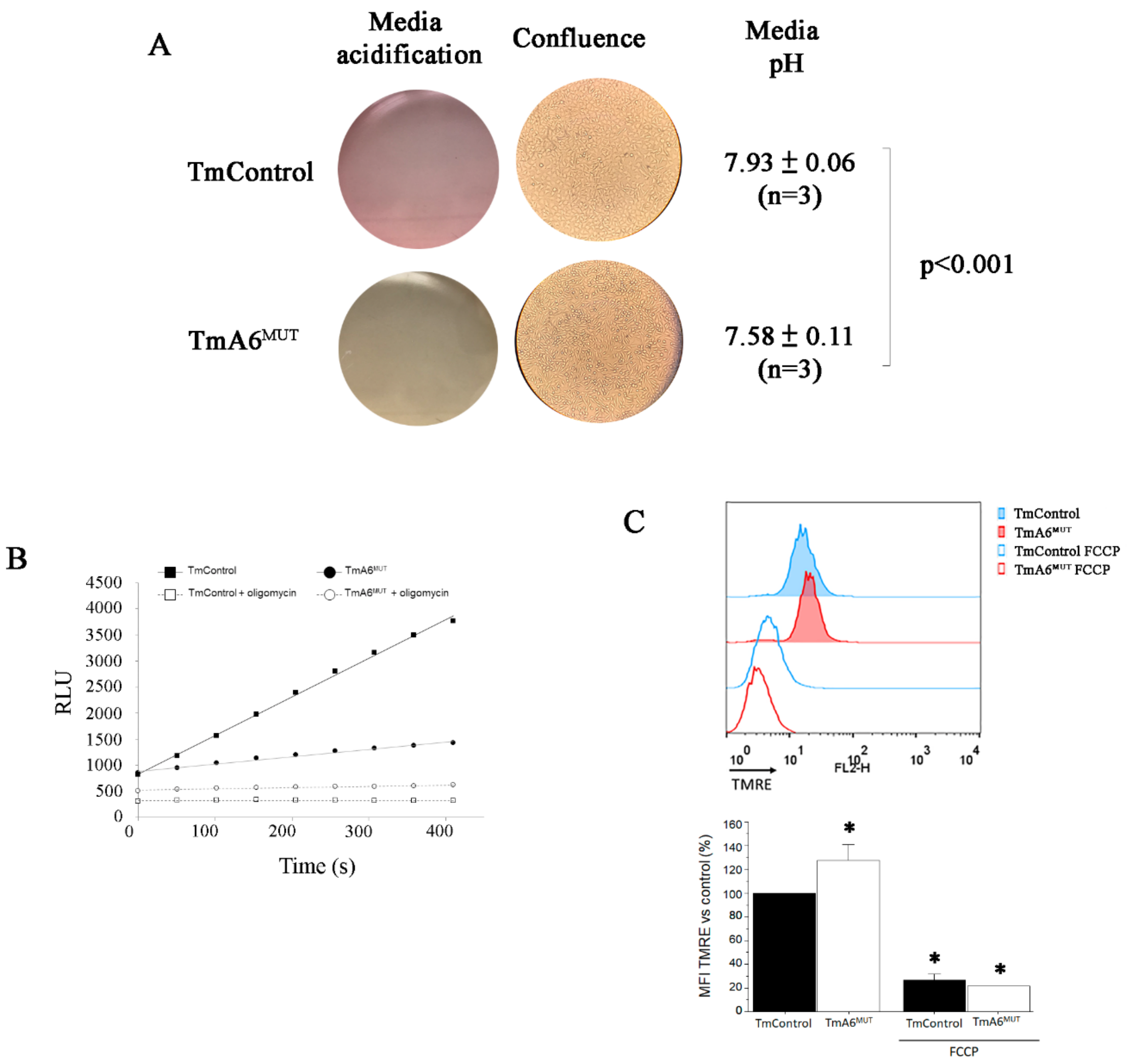

Figure S1. Supplemental OXPHOS phenotype characterization. **A)** Culture medium acidification differences (left panel) for the same cell density (middle panel) in TmControl and TmA6^{MUT}cells. **B)** Linear luminiscence curve corresponding to ATP synthesis activity obtained from $2x10^6$ digitonin permeabilized control and mutant cells, in the presence or absence of 0.5 µg of oligomycin. Luminiscence (relative light units, RLU) was recorded at 50 s intervals. **C)** $\Delta\Psi m$ evaluation by flow cytometry after TMRE staining.The upper panel shows a representative histogram of $\Delta\Psi m$ measurement in both cell lines under basal conditions and after treatment with FCCP to uncouple oxygen consumption and ATP synthesis. The lower panel represents the average $\Delta\Psi m$ of mutant cells relative to controls from two independent experiments. Graph represents the mean ± S.D., asterisks indicate significant difference, tested by ANOVA post-hoc Fisher PLSD ($p<0.05$), with respect to TmControl without uncoupler.

Table S2: Figure 2B Data: Glucose consumption and lactate production by TmControl and TmA6^{MUT} cells

Cell Line	Sample	[Lactate] (mM)	[Glucose] (mM)	[Glucose]mean (mM)
TmControl	1	3.401	9.303	
TmControl	1	3.16	11.772	10.538
TmControl	2	4.001	9.127	
TmControl	2	4.919	10.891	10.009
TmControl	2	3.086	---	
TmControl	2	3.423	---	
TmA6MUT	1	8.055	17.416	
TmA6MUT	1	7.352	19.885	18.651
TmA6MUT	2	7.293	19.004	
TmA6MUT	2		20.944	19.974
TmA6MUT	3	7.226	20.062	
TmA6MUT	3	8.18	19.533	19.797

Table S3: Figure 2C Data: Endogenous and maximal oxygen consumption rate measurement in intact cells

Cell Line	Endogenous respiration (fmol O_2/min/cell)	Maximal respiration (fmol O_2/min/cell)
TmControl	3.64	8.55
TmControl	3.31	8.07
TmControl	3.39	7.44
TmControl	3.25	6.58
TmControl	4.95	11.24
TmControl	6.12	14.23
TmControl	2.95	4.74
TmControl	4.74	6.97
TmControl	3.33	5.3
TmA6^{MUT}	1.09	2.09
TmA6^{MUT}	0.92	1.84
TmA6^{MUT}	1.47	4.68
TmA6^{MUT}	1.33	5.37
TmA6^{MUT}	1.38	3.97
TmA6^{MUT}	1.81	6.95
TmA6^{MUT}	2.04	8.19
TmA6^{MUT}	1.69	6.53
TmA6^{MUT}	2.67	8.19
TmA6^{MUT}	2.20	8.11
TmA6^{MUT}	2.54	9.49
TmA6^{MUT}	2.69	4.47
TmA6^{MUT}	2.49	3.66

Table S4: Figure 2D Data: Oxygen consumption of digitonin-permeabilized cells

Cell Line	CI+III+IV ($fmolO_2$/min/cell)	CII+III+IV ($fmolO_2$/min/cell)	CIV ($fmolO_2$/min/cell)
TmControl	.	5.79	16.24
TmControl	2.84	3.97	10.51
TmControl	3.14	4.31	14.47
TmA6^{MUT}	1.36	1.95	7.64
TmA6^{MUT}	1.37	1.95	7.64
TmA6^{MUT}	0.78	1.13	5.73

Table S5: Figure 2E Data: DCA and Iodoacetate IC_{50}

Cell Line	IC_{50} DCA (% of TmControl)	IC_{50} IA (% of TmControl)
TmControl	93.9	96.2
TmControl	106.1	117.7
TmControl	100.0	86.1
TmA6^{MUT}	41.9	62.4
TmA6^{MUT}	39.3	71.9
TmA6^{MUT}	32.3	87.4
ρ^0	55.3	33.5
ρ^0	62.5	45.3
ρ^0	---	38.4

Table S6: Figure 3A Data: ATP synthesis rate

Cell Line	ATP synthesis rate (% of control)
TmControl	85.7
TmControl	126.1
TmControl	88.2
TmControl	116.7
TmControl	83.3
TmControl	100.6
TmControl	99.4
TmControl	101.8
TmControl	98.2
TmA6^{MUT}	18.4
TmA6^{MUT}	17.6
TmA6^{MUT}	15.8
TmA6^{MUT}	14.0
TmA6^{MUT}	0.6
TmA6^{MUT}	0.7

Table S7: Figure 3B Data: Oligomycin sensitivity

Cell Line	IC_{50} Oligomycin (nM)	IC_{50} Oligomycin (% of control)
TmControl	157.1	102.6
TmControl	196.8	128.6
TmControl	120.3	78.6
TmControl	137.8	90.1
TmA6^{MUT}	61.4	40.1
TmA6^{MUT}	22.7	14.8
TmA6^{MUT}	31.5	20.5
TmA6^{MUT}	38.0	24.8

Table S8: Figure 3C Data: ΔΨm measurement

Cell Line	$DiOC_6$ MFI (% of Control)
TmControl	100.0
TmControl	100.0
TmA6^{MUT}	151.0
TmA6^{MUT}	112.0

Table S9: Figure 4A Data: mtROS measurement

Cell line	MitoSOX MFI (% of Control)
TmControl	100
TmControl	100
TmA6^{MUT}	126
TmA6^{MUT}	135

Table S10: Figure 4B Data: Mitochondrial Mass evaluation

Cell line	MitoTracker Green MFI (% of Control)	mtDNA copy number (% of Control)
TmControl	100	108.2
TmControl	100	99.6
TmControl		92.2
TmControl		90.7
TmControl		109.3
TmA6^{MUT}		29.1
TmA6^{MUT}	64	33.6
TmA6^{MUT}		24.0
TmA6^{MUT}		32.6
TmA6^{MUT}		57.6

Table S11: Figure 4C Data: mtROS/mt mass

Cell line	mtROS/mtMass
TmControl	100.0
TmControl	100.0
TmControl	100.0
TmA6^{MUT}	213.6
TmA6^{MUT}	210.9
TmA6^{MUT}	136.6

Table S12 Figure 4D Data: Endogenous respiration in intact cells vs mt mass

Cell line	End. Resp. vs mt
TmControl	3.64
TmControl	3.31
TmControl	3.39
TmControl	3.25
TmControl	4.95
TmControl	6.12
TmControl	2.95
TmControl	4.74
TmControl	3.33
TmA6^{MUT}	2.45
TmA6^{MUT}	2.22
TmA6^{MUT}	2.30
TmA6^{MUT}	3.02
TmA6^{MUT}	3.40
TmA6^{MUT}	2.82
TmA6^{MUT}	4.45
TmA6^{MUT}	3.67
TmA6^{MUT}	4.23
TmA6^{MUT}	4.15
TmA6^{MUT}	1.82
TmA6^{MUT}	1.53

Table S13 Figure 4E Data: Oxygen consumption rate in digitonin permeabilized cells vs mt mass.

Cell line	CI+III+IV vs mt mass	CII+III+IV vs mt mass	CIV vs mt mass
TmControl	---	5.79	16.24
TmControl	2.84	3.97	10.51
TmControl	3.14	4.31	14.47
TmA6^{MUT}	2.21	3.17	12.42
TmA6^{MUT}	2.23	3.17	12.42
TmA6^{MUT}	1.27	1.84	9.32

Table S14 Figures 6B & D Data: MHC-I analysis.

Cell Line	[DCA] (mM)	MHC-I vs control	MHC-I vs No drug
TmControl	0	1.00	1.00
TmControl	5	1.32	1.32
TmControl	25	1.49	1.49
$TmA6^{MUT}$	0	2.08	1.00
$TmA6^{MUT}$	5	2.43	1.17
$TmA6^{MUT}$	25	2.52	1.21

Table S15 Figures 6C Data: % of cell death and growth after DCA treatment.

Cell Line	[DCA] mM	% of Apoptosis	% of Cell Growth (MTT)
TmControl	0	1.1	100.8
TmControl	0	2.3	99.2
TmControl	0	4.5	
$TmA6^{MUT}$	0	4.9	102.4
$TmA6^{MUT}$	0	4.3	97.6
$TmA6^{MUT}$	0	5.7	
TmControl	5	1.4	101.1
TmControl	5	4.9	106.0
TmControl	5	7.4	
$TmA6^{MUT}$	5	8.7	78.0
$TmA6^{MUT}$	5	5.6	79.7
$TmA6^{MUT}$	5	6.4	
TmControl	25	23.5	103.4
TmControl	25	6.8	110.9
TmControl	25	12.1	
$TmA6^{MUT}$	25	20.2	57.9
$TmA6^{MUT}$	25	41.1	49.4
$TmA6^{MUT}$	25	59.7	

Table S16 Figures 6E Data: mtROS production after DCA treatment

Cell Line	[DCA] mM	MitoSOX vs No drug
TmControl	0	100
TmControl	5	87
TmControl	25	144
$TmA6^{MUT}$	0	100
$TmA6^{MUT}$	5	110
$TmA6^{MUT}$	25	314

Figure 6H Data: The datasets generated to analyse cell migration are too big to be published in this Supplementary Material file but they will be available from the corresponding author on reasonable request.
